# How blogs support the transfer of knowledge into practice in the field of dementia palliative care: a survey of facilitators and barriers

**DOI:** 10.1186/s12904-022-01001-7

**Published:** 2022-07-01

**Authors:** Aphie Rukundo, Siobhan Fox, Suzanne Guerin, George Kernohan, Jonathan Drennan, Niamh O’Connor, Suzanne Timmons

**Affiliations:** 1 Present Address: Centre for Gerontology and Rehabilitation, School of Medicine, University College Cork, Cork, Ireland; 2 School of Psychology, University College Dublin, Dublin, Ireland; 3 School of Nursing and Paramedic Science, Ulster University, Belfast, Northern Ireland; 4 School of Nursing and Midwifery, University College Cork, Cork, Ireland

**Keywords:** Dementia, Palliative Care, Knowledge Transfer, Dissemination, Social Media, Blogs, Education

## Abstract

**Background:**

Blogging can help to maximise the impact of one’s work in academia and beyond by making research findings accessible for multiple knowledge users, such as healthcare professionals and the public, as well as other researchers. As part of the knowledge exchange and dissemination activities of the Model for Dementia Palliative Care Project, this study explored stakeholders’ views of blogs as a means to translate research findings.

**Methods:**

A web-based survey was developed, piloted, and revised. It was distributed electronically via key dementia and palliative care organisations websites, newsletters, social media platforms, and within the staff mailing lists of five Universities in Ireland. Data were analysed using descriptive statistics and content analysis.

**Results:**

Complete responses were received from 128 participants. The majority of respondents were healthcare researchers (*n* = 53), followed by healthcare providers (*n* = 46). The preferred methods of reviewing research findings were scientific papers, websites and news articles. Respondents read healthcare blogs “sometimes” (39.1%), with < 19% reading them “often” or “very often”. Receiving an email notification might increase the likelihood of reading a new blog post for 83% of respondents. Barriers to engaging with blogs included lack of time, preference for other media, lack of awareness regarding available blogs, and concerns about the credibility and source of information. An appropriate length and the author of the blog were key features that encouraged engagement with a blog.

**Conclusions:**

Despite respondents choosing a scientific paper as their preferred method to consume research findings, many indicated an openness to reading blogs on their area of interest. Creating concise, relevant, and credible blogs, and suitably promoting them, could increase the impact and reach of healthcare research, such as in the emerging field of dementia palliative care, and thus promote translation of research findings into practice.

**Supplementary Information:**

The online version contains supplementary material available at 10.1186/s12904-022-01001-7.

## Background

### The clinical context of dementia

The number of people living with dementia is estimated to be in excess of 55 million worldwide, and this is increasing. Dementia causes people to experience a range of cognitive and non-cognitive symptoms [1]. Although some symptoms can be alleviated by medication, there is no single curative treatment available for dementia. Thus, all treatment is essentially palliative [2].

Palliative care is appropriate for anyone with a life-limiting illness including Dementia [3]. Despite growing international recognition, people living with dementia still receive poor palliative care including end-of-life care [4]. A review of the factors influencing provision of palliative care services for people with dementia found that barriers stem from a largely limited knowledge base and lack of awareness amongst healthcare staff that dementia is a terminal illness and therefore requires a palliative care approach after diagnosis [5, 6].

### Knowledge transfer in palliative dementia care

As the growing international literature illustrates the benefits to be experienced from adopting a palliative care approach to dementia, it is imperative to implement methods of knowledge transfer and dissemination to apply knowledge to practice [7, 8]. The Evidence-based Model for Transfer & Exchange of Research Knowledge (EMTReK; [9]) was developed to apply learning from existing knowledge transfer exchange (KTE) models to palliative care research. The model outlines core elements for successful knowledge transfer, including: 1) the key messages need to be important to the user, easily accessible and credible; 2) the key stakeholders must be identified to allow interactive exchange, including knowledge users (doctors, nurses, etc.), knowledge beneficiaries (the service users; the public) and knowledge producers (researchers); and 3) the processes or mode of dissemination must reflect the target audience. The model highlights that ‘marketing’ one’s research findings increases the likelihood of transferring research knowledge into practice. Hence, social media presents a remarkable opportunity for effective KTE [10, 11].

Although there is limited evidence on social media's impact on health policy [12], the potential to promote dissemination of evidence-based literature is increasingly evident [11]. Cheston et al.’s systematic review [13] found that interventions using social media tools were associated with improved knowledge, attitudes, and skills, as well as promoting learner engagement, collaboration, feedback, and professional development. A cross-sectional study assessing the use of social media amongst healthcare professionals found that a substantial percentage (91.9%) of healthcare professionals used social media networks, with a sizeable proportion of doctors and nurses utilising this for work-related tasks such as medical reading, and engaging in online medical forums [14]. Similarly, the use of social media in medical education for physicians and physicians-in-training has proliferated.

### Acquiring up-to-date knowledge online

A recurring barrier to professional development and education amongst healthcare staff in the literature is time constraints [15, 16]. With different shifts, busy wards and patient acuity, staff often report feeling under pressure at work, leaving them with no time for peer-to-peer knowledge sharing. Blogs have also been proposed as a medium to develop a community through information sharing. In a case study of medical students attending a conference and blogging about the presentations attended [17], results revealed an augmented sharing of information through online discussion forums and a development of a community of learners who were engaged in the conference, even if not attending. In another study [18], an online survey was administered to nurses (*N* = 37) in a cardiac intensive care unit to explore perceptions of blogs as a forum for professional education. Responses obtained 15 months after the implementation of these blogs indicated that most participants thought they were an effective way to share professional education (86%) and keep them aware of recent evidence-based practice (81%). Furthermore, 59% believed this led to practice change, with 62% stating they would consider contributing to blog posts as a result.

As blogging becomes increasingly accepted by the healthcare professional community, it is important to identify the facilitators and barriers to engaging with blogs within this target population. As a method of knowledge transfer, blogs may be particularly useful in the area of dementia palliative care where there are persistent misconceptions and lack of awareness about the topic [8]. Therefore, the aim of this study was to investigate the opinions of healthcare providers and researchers, and other stakeholders’ opinions on blogs. For the purpose of this study, a blog is defined as a regular feature appearing as part of an online publication that typically relates to a particular topic and consists of articles and personal commentary by one or more authors. The two specific research questions were: What are the current views of healthcare providers, health service researchers, and other stakeholders on blog posts as a means of translating research findings, and what are their preferences for accessing and reading blog posts?

## Methods

### Survey development

Building upon the Evidence-based Model for Transfer & Exchange of Research Knowledge (EMTReK; [12]), a 14-item survey was developed and delivered on an online survey platform. The survey was piloted with a convenience sample of healthcare providers and researchers (*N* = 6) and revised and re-piloted (*N* = 2) before being widely distributed. Pilot data were not included in the final data. Beginning with a definition of a blog (for clarity), the survey had three sections: participants’ demographics; participants’ views and opinions about blogs in general (using a mixture of question types); and the specific features of blogs that may affect respondents’ engagement (see supplemental data for final survey).

### Survey administration

The survey was targeted to four groups: healthcare providers and managers; healthcare researchers; policy makers; and ‘others with an interest in healthcare research’. Thus, the first survey question listed these options along with a “none of the above” option, which excluded participants from the survey. This was the only inclusion/exclusion criteria for the sample.

The survey was advertised via the websites, newsletters and social media pages of key national organisations related to dementia and/or palliative care (e.g. the All Ireland Institute of Hospice and Palliative Care, the National Dementia Office, Dementia and Neurodegeneration Network Ireland, etc.), and via the staff mailing lists of five universities in the island of Ireland.

### Data analysis

Descriptive statistics were used to elicit any trends present in the data. The data set did not meet normality for key data, so nonparametric tests were conducted to test between-group differences, (IBM-SPSS version 22). Open-ended responses to survey items were analysed using content analysis.

## Results

### Survey respondents

The number of people who received the survey is not known due to the recruitment process. After discarding responses with blank or incomplete data, 128 replies were received, from healthcare researchers (*n* = 53), healthcare providers and managers (*n* = 46) and others with an interest in healthcare research (*n* = 29). This latter are henceforth referred to as “interested other”. As the number of healthcare managers was small (*n* = 9), this group was merged with healthcare providers (*n* = 37); both groups had similar response patterns identified by running descriptive statistics on key outcome measures. No respondents identified themselves as policy-makers. Reflecting the recruitment process, most of the sample were from Ireland (*n* = 111). Nearly 72% of respondents were female. Most respondents were aged < 45 (Table 1).

**Table 1:** Respondent demographics (*N* = 128) showing the range of respondent characteristics

	**N**	**%**
**Primary Occupation**		
Healthcare researcher	53	41.4
Healthcare provider/manager	46	35.9
Other with an interest in healthcare research	29	22.7
**Age**		
≤ 34	35	27.3
35–44	35	27.3
45–55	31	24.2
≥ 56	21	16.4
Missing	6	4.7
**Gender**		
Female	92	71.9
Male	26	20.3
Prefer not to identify	2	1.6
Missing	8	6.3
**Country**		
Ireland	111	86.7
Northern Ireland	5	3.9
UK	2	1.6
Australia	1	0.8
Canada	1	0.8
Missing	8	6.2

### Opinions about blog posts, in general and relating to healthcare and/or research

Participants’ preferred sources for healthcare knowledge were explored by ranking stated options (1–8). Combining the top 3 rankings (i.e. where the option was ranked in the top three by a respondent), a scientific paper was the preferred source at 73.9%, followed by a news article at 57.5% and website at 51.9%. Blogs came fifth in this analysis at 28.3% occurrence in the top three rankings, below tweets (33.9%; see Table 2).

**Table 2:** Preferred source for healthcare knowledge showing the dominance of scientific papers

	**Blog Article**	**Scientific Paper**	**Poster**	**News Article**	**Podcast**	**Video**	**Tweet**	**Website**
1st preference	5.5%	47.2%	1.6%	12.6%	5.5%	3.9%	13.4%	10.2%
2nd preference	13.4%	10.2%	8.7%	18.1%	6.3%	7.9%	7.1%	28.3%
3^rd^ preference	9.4%	16.5%	3.1%	26.8%	12.6%	4.7%	13.4%	13.4%
Occurrence in top 3 rankings	28.3%	73.9%	13.4%	57.5%	24.4%	16.5%	33.9%	51.9%

The open text responses provided insights into possible reasons for the low preference for blogs over other methods of acquiring knowledge. Respondents wanted to be assured that articles are produced by a trusted author that is knowledgeable in the area (It would depend on who the author of the blog is, their knowledge of the topic and their conflicts of interest*,* Participant 102, healthcare provider) and that content is written with rigour and backed up with scientific evidence ([blogs are] basically science journalism, without the oversight of a publisher, etc*.,* Participant 16, healthcare researcher). The impact of these issues is captured in the following quote:

Usually, I just scroll to the bottom [of the blog] to find the reference to the scientific paper. I do occasionally read them if I am interested in the opinion of the author specifically, but I am usually concerned about inappropriate reporting of research results (quackery or poor interpretation of basic science). (Participant 75, interested other).

### Frequency of reading blogs in general and relating to healthcare and/or research

Respondents’ engagement with blogs was explored with the question “How often do you read blog posts (about any topic)?”. The most popular option (39.1%) was that the participant read blog posts “sometimes”. This was closely followed by 29.7% of participants who *“*rarely” read blogs, while 12.5% “never” reading blogs. When probed specifically on reading healthcare blogs, responses were overall similar, with 39.1% “sometimes” reading healthcare blogs, 33.6% “rarely”, and 11.7% “never” doing so.

Statistical analyses were conducted to explore any existing relationships between variables. Multiple comparisons are not included because the overall tests did not show significant differences across samples.

A Kruskal–Wallis Test revealed no significant difference in all blog reading levels across the four different age groups χ2 (3, *n* = 122) = 2.83, *p* = 0.419. An additional analysis exploring the relationship between healthcare blog reading levels across the four age groups was also conducted. No significant differences were found. χ2 (3, *n* = 121) = 2.83, *p* = 0.728.

Similar statistical analyses were run to examine any differences in regard to blog reading across different occupation groups. A Kruskal–Wallis Test revealed a non-statistically significant difference in blog reading levels across the different occupation groups, χ2 (2, *n* = 126) = 2.78, *p* = 0.249. This trend remained in healthcare blog reading levels across occupation groups: χ2 (2, *n* = 125) = 1.07, *p* = 0.585.

### Willingness to read dementia palliative care blogs

Participants’ willingness to read dementia palliative care blogs was explored via question 12, “If we were to conduct monthly blog posts in the area of dementia palliative care research, would you read them?”. The majority of responses were positive with 60.7% of the sample being open to reading them (37.7% ‘yes’ and 23.0% ‘maybe’).

### Factors increasing blog engagement

Further probing with the question “If you answered “rarely” or “never” to question 6, would you consider reading blog posts relating to research projects if they were made available to you?”, indicated that 82.9% of these respondents (n = 70) would consider reading blogs if an email notification was sent to them. Similarly, across the whole sample, 82.8% indicated they might be more likely to read a blog post (57.8% ‘more likely’ and 25% ‘perhaps more likely’) if they received an email notification.

Further insight into these responses were evident across the qualitative data, where accessibility was a common barrier cited by respondents to using blogs as a source of healthcare knowledge. Participants commented that.

Sometimes the websites that these blogs are available on are not easy to find*.* (Participant 97, healthcare researcher).

Accessibility is key, when articles are readily available it makes things a lot easier (Participant 120, healthcare provider).

In addition to accessibility, participants also noted a lack of knowledge of good quality blogs:

I just don’t know any good blogs in regard to research*.* (Participant 150, interested other).

Would depend on if it was easy to find the posts and if the topics were interesting (Participant 90, healthcare researcher).

Thus, sending an email notification with the blog article attached was highlighted to have high potential in increasing participants’ likelihood of engaging with blogs to address both accessibility and awareness.

Qualitative responses also illustrate that respondents’ current lack of engagement with blogs is a passive circumstance and not an active decision.

My lack of use probably relates more to habit than to any fixed decision (Participant 122, healthcare provider).

The only blogs I subscribe to at present are from the regulatory agencies like MHRA in UK, HPRA in Ireland. I am not aware of blogs on therapeutic areas. If I saw a twitter post or got email about one, I might follow it and read the blog (Participant 116, healthcare researcher).

**Fig. 1:**
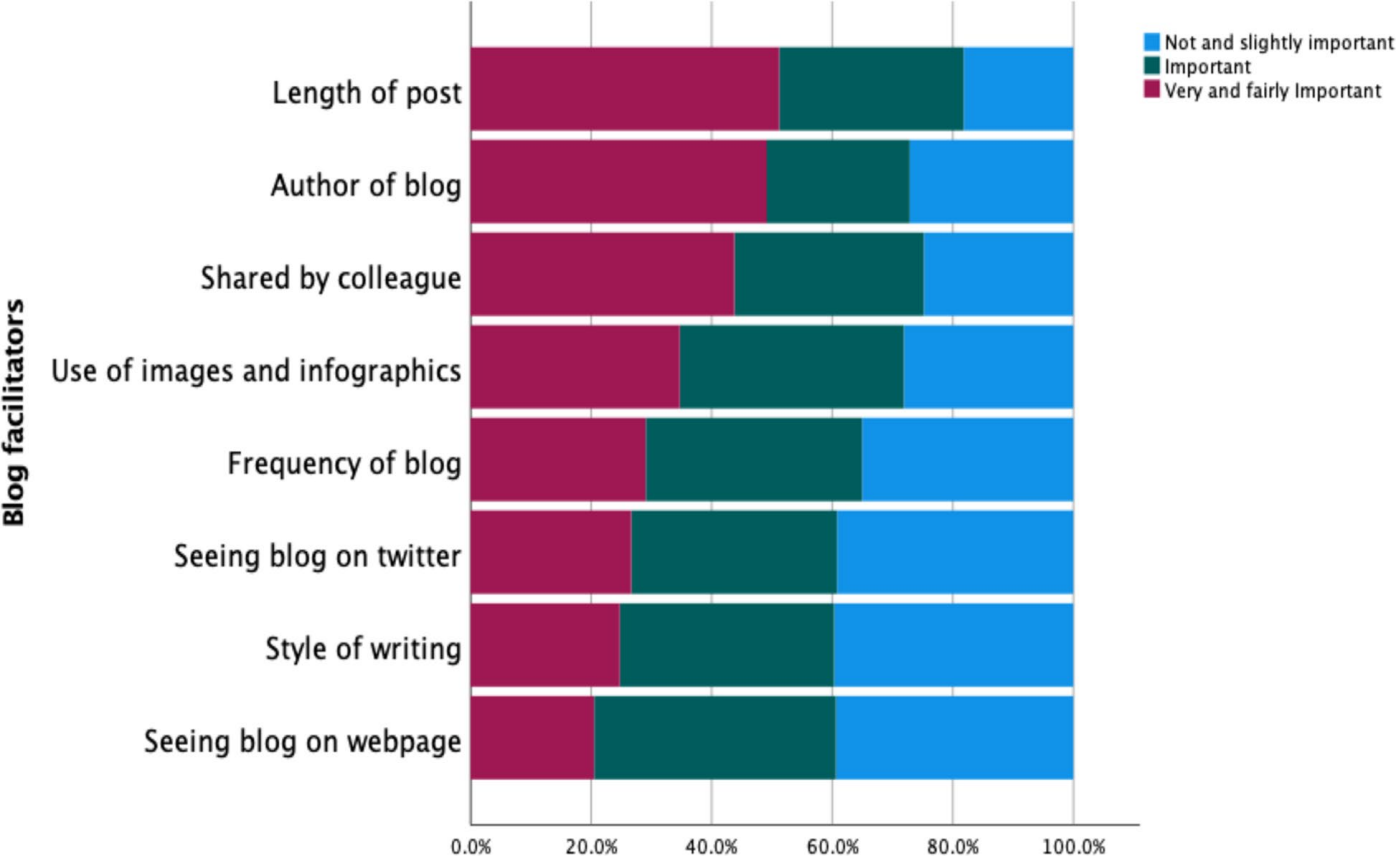
Aspects of blogs considered important by readers in descending order

A further question asked respondents to rate the importance of eight factors that have the potential to facilitate their engagement with blogs, using a 5-point Likert scale (Fig. 1). The blog length, author, seeing a colleague share the blog, and use of images/infographics were most important to respondents.

**Fig. 2:**
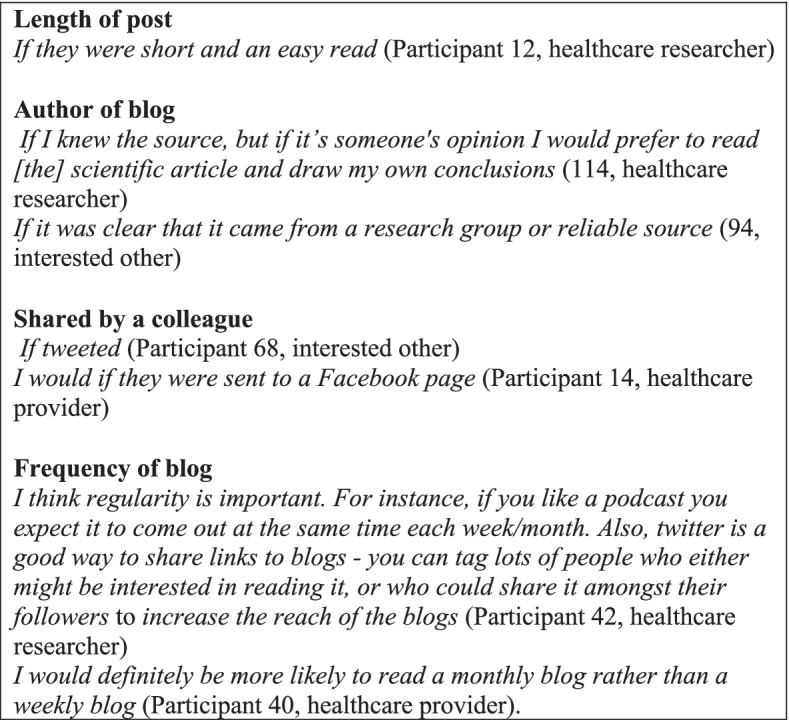
Qualitative quotes showing the importance of editorial and promotional attention

The style of writing was the feature most often chosen as “Not important”. Similar views were shared in the qualitative responses, with illustrative quotes included in Fig. 2.

Looking descriptively at data from the three occupation groups (healthcare providers, healthcare researchers, interested other) they provided similar findings with the blog length and author taking a spot in the top three positions under “Important*”*. A blog being shared by a colleague was of greater importance to healthcare providers whereas the use of infographics was predominant for healthcare researchers and style of writing took precedence for interested others.

### Barriers to engaging with blogs

**Fig. 3:**
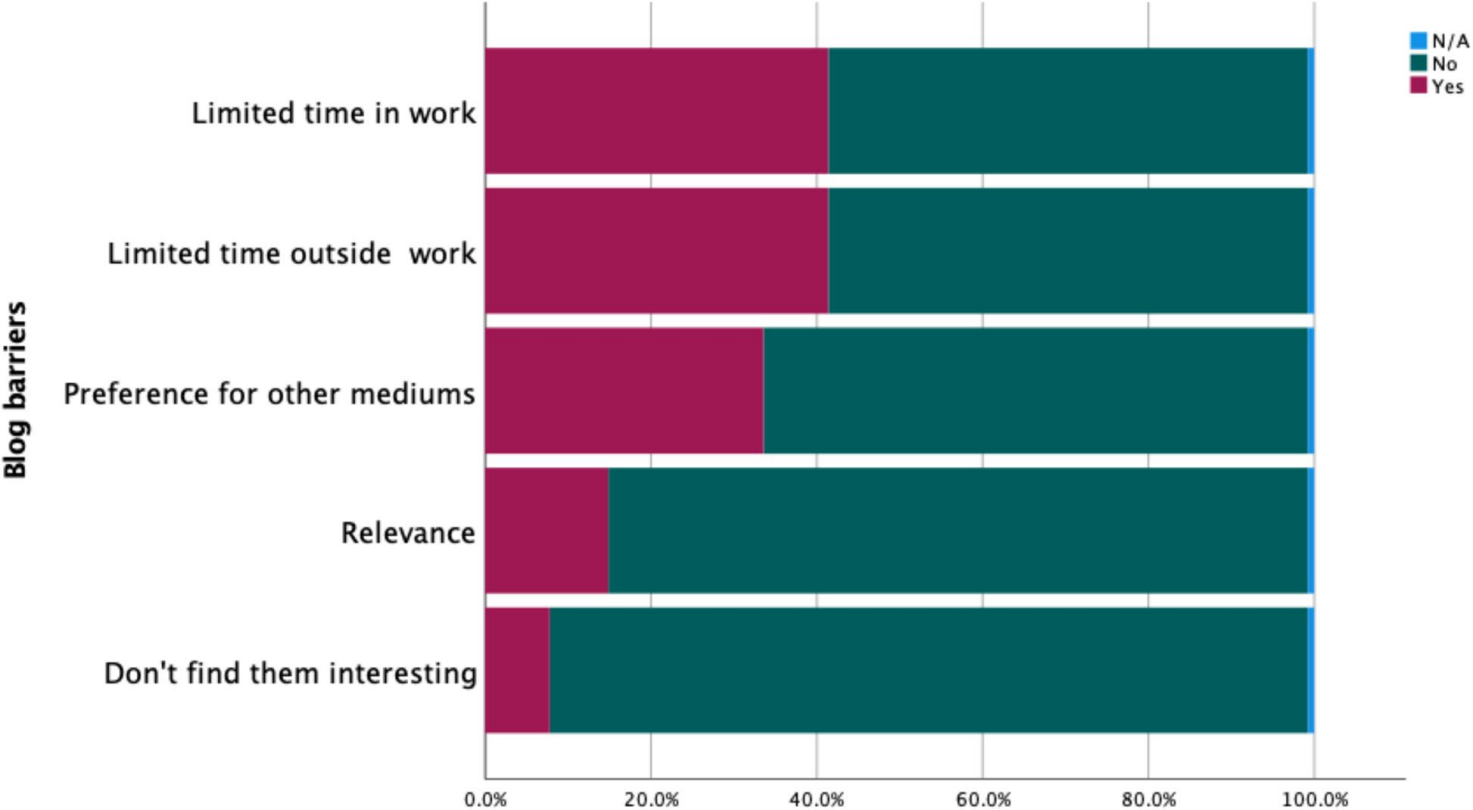
Blog barriers in descending order

Barriers are defined as features that would present as an obstacle to participants reading blogs for their healthcare knowledge. Five potential barriers, presented to participants, were ranked as follows (Fig. 3)*.*

Lack of time to prioritise blogs was prominently cited by respondents in qualitative responses. This included not having enough time at work, and not having enough time to devote to blogs out of work (See Fig. 3).

Reading the blog would depend on the title & if I had enough time in my day (Participant 67, healthcare provider).

[I] often don't have time to go searching for all the different things I need to or want to read (Participant 59, healthcare manager)

Related to limited time, respondents indicated only having the opportunity to read blogs which are most highly relevant or interesting to them:

While I am interested in Palliative Care, I am so busy I cannot commit to reading much outside my area of specialty (oncology) (Participant 100, healthcare researcher).

It would depend on my available time and how interesting or relevant the posts were to my own work (Participant 91, healthcare researcher)

Other barriers preventing participants from reading blogs included doubts regarding their scientific integrity and an unawareness of the existence of these blogs and/or how to access them.

My main issue with blogs is in relation to the quality of them, establishing the validity of data presented, and who is developing them, basically quality control (Participant 83, healthcare researcher).

Difficulty finding/ locating new blogs to read (Participant 101, healthcare provider)

A preference for other media was also often cited.

I tend to not take blogs as seriously as I would a scientific paper or poster or what I may read from an accredited website associated with the research. (Participant 89, healthcare researcher).

I'd prefer to await a robust academic paper (Participant 33, healthcare researcher).

## Discussion

### Summary of results

The aim of this study was to explore the attitudes of healthcare providers/managers, healthcare researchers, and others with an interest in research in the dementia and palliative care field, to blogs, including their preferences for different blog features and the effectiveness of blogs as a method to disseminate research findings. To the best of our knowledge, this is the first study to explore participants’ willingness to read dementia palliative care blogs, where the majority of respondents would consider reading dementia palliative care blogs but currently only read blogs ‘sometimes’ or ‘rarely’. However, blogging still has the potential to be an effective method of knowledge sharing amongst these cohorts, if certain criteria are met.

Reflecting the core elements of the EMTReK model [9], the main facilitators identified herein included easy accessibility, a credible source, a colleague sharing the blog, and adequate ‘marketing’. Respondents, across all occupations and ages, perceived time as the most persistent barrier to engaging with blogs, with insufficient time in work and outside work to engage with blogs. A lack of time is a common barrier to many processes in the workplace, both in healthcare and research.

### Increasing blog reach and engagement

Organisations must make an active effort to ensure employees have the resources (especially time) available to access and make use of evidence based research, be that via blogs or other means. Our research also suggested that blog’ reach and engagement could be improved by an appropriate length, frequency and regularity of the blog posts, and by advertising of the blog, by word of mouth from colleagues, and by email notifications. A summary of recommendations is presented in Table 3.

**Table 3:** Recommendations to increase blog reach and engagement

1. Identify a credible source (university, researchers, qualified author, expert by experience, etc.)
2. Use short and concise blog posts
3. Provide an estimated reading time
4. Follow a regular and consistent posting schedule (e.g. 1 per month)
5. Disseminate blogs on social media platforms: e.g. Twitter, LinkedIn, Facebook groups
6. Promote blog through relevant organisations internally
7. Provide an email notification for new posts
8. Give allocated time at work for informal knowledge enhancement including reading blogs
9. Moderate any blog comment section to avoid “fake news” and misinformation

It is interesting to compare the current findings to the small existing literature on the use of blogs to promote knowledge exchange in healthcare research and practice. Our findings diverge from the study by Khan et al. [14] where 46% of healthcare staff used social media (not just blogs) for work-related tasks such as medical reading, and 11% engaged in online medical forums. It must be noted that the latter was conducted in America where perhaps different policies are in place regarding the use of social media at work. A study conducted on the facilitators and barriers of KTE in a palliative care setting in Ireland has findings aligned with the current study, with inadequate time as a core theme in barriers to KTE [10]. This suggests that for blogging to be effective in knowledge dissemination, a systemic change may be required at the level of workplace policies to ensure people interested in reading blogs relevant to their work have the time to do so.

Furthermore, the positive responses to websites and tweets as sources of healthcare knowledge is notable. To increase engagement with blogs, they could be posted on reputable websites and promoted via Twitter. Our study indicates that Twitter has more influence than posting on a website, which makes sense as the recipient has to actively go to a website to seek a blog, whereas a tweet brings them the link to the site. In addition, the recommendation of colleagues is important, which might be via twitter, email or verbally. Indeed, an email notification was perceived to be a powerful facilitator of engaging with a blog, even by those who seldom read blogs currently. This finding emphasises the growing role of social media as a knowledge dissemination tool and its potential in meeting the knowledge marketing component in the EMTReK model [12]. In simple terms, a blog may be insightful, educational etc., but if few people know of it, its reach may be limited.

### Application to dementia palliative care

The current results highlight the importance of considering the target audience when writing and promoting a healthcare blog, as different styles of blogs suit different audiences. Results suggested that healthcare providers may be influenced by a colleague sharing the link, whereas for researchers, the use of infographics mattered more. The type of language used, i.e., accessibility, was highly valued for non-professionals interested in healthcare. This should prompt healthcare blog authors and researchers to think of all the different stakeholders and attributes required to effectively transfer knowledge into practice.

As dementia palliative care continues to be widely misunderstood in society, it is important that efforts are made to destigmatize, educate and increase awareness among healthcare workers and researchers and also in the wider public. Blogging has the potential to meet the varied components of effective knowledge transfer, supported by a dissemination model designed with palliative care in mind (EMTReK). Blogging on dementia palliative care meets the three core elements of the EMTRek Model, as follows: it addresses a recognised knowledge gap; blogs are easily accessible to the three stakeholder groups (knowledge-users, -beneficiaries, and -producers) and allows interactive, open discussions; and the growing popularity of social media use supports blogs as a potentially time-effective and easier knowledge marketing medium**.**

### Limitations

Although the survey tool was created by a group of experts and subsequently piloted twice, it is not yet a fully validated tool. The survey respondents are mainly from an Irish population and may not be generalizable to other countries. The study also had a relatively small sample size which limits the potential for definite inferences to be made.

## Conclusion and implications for future research

There is a scarcity of research addressing the use of blogging in healthcare knowledge transfer and exchange, despite its documented explosive growth in the social arena. This study explores the use of blogs as a potential medium to translate dementia palliative care research into practice, as an emerging field that requires multiple partners across health and social care and the wider society, and where many misconceptions persist, such that quickly reaching a wide audience with simple, clear messages is important. This study found that scientific papers remain the preferred source for healthcare knowledge, and healthcare/research blogs are infrequently read. Recommendations to increase the reach of healthcare blogs include easy accessibility, a credible source and effective marketing. With time constraints as the main barrier to be addressed, blogs should be easy to find, and short and concise while maintaining the key messages. Additional research may further elucidate patterns specific to age or occupation that can in turn be used to adequately target specific groups.

## Supplementary Information

**Additional file 1.** Supplementary material.

## Data Availability

The datasets used during the current study are available from the corresponding author on reasonable request.
