# Supplementary material for: How blogs support the transfer of knowledge into practice in the field of dementia palliative care: a survey of facilitators and barriers
Source: BMC Palliat Care. 2022 Jul 1;21:117. doi: 10.1186/s12904-022-01001-7 (PMC9248102; doi:10.1186/s12904-022-01001-7)
Supplement: Supplementary file 1 — Additional file 1. Supplementary material. [file 12904_2022_1001_MOESM1_ESM.pdf]

How blogs support the transfer of knowledge into practice in the field of dementia palliative care: a survey of facilitators and barriers

Definition of a blog for the purpose of this research:

*A **blog** is a regular feature appearing as part of an online publication that typically relates to a particular topic and consists of articles and personal commentary by one or more authors.*

Section one: About you

1. **Please confirm the primary area/discipline of your work:**
   (Options: healthcare provider; healthcare manager; healthcare researcher; policy-maker; other with an interest in healthcare research (please state); none of the above, <u>if none</u> -> exit survey)

2. **Gender**
   (Options: Male/ Female/ Other/ Prefer not to identify)

3. **What age group are you?**

   (Options: ≤24 / 25-34 / 35-44 / 45-55 / 56-65 / ≥66)

4. **Do you write blogs in your line of work to convey information?**

   (Options: Yes, often / Yes, occasionally / No, never)

Section two: Views and opinions about blogs in general

5. **How often do you read blog posts (about any topic)?**
   (Options: Very often / Often / Sometimes / Rarely / Never)

6. **How often do you read blog posts relating to healthcare and/or research?**

   (Options: Very often / Often / Sometimes / Rarely / Never)

7. **If you answered "rarely" or "never" to question 6, would you consider reading blog posts relating to research projects if they were available to you?**
   (Option: Yes/Maybe/No)

   Please explain your response?

   ____

8. **Would you be more likely to read/listen to information ONLINE about research in the format of:**

(Options: Rank 1-8, 1 being most likely, 6 being least likely)

- A blog post
- A scientific paper
- A poster
- News article
- Podcast
- Video
- A tweet
- A website

9. Would getting an email notification when a new blog post is posted make you more likely to read a blog post?
(Option: Yes/ No/ Maybe)

10. What barriers are there to you reading blogs about healthcare / research (please tick as many as apply)?

(a) Don't find them interesting
(b) Don't have enough time at work
(c) Don't have enough spare time outside of work
(d) Existing blogs are not relevant to me
(e) I prefer other mediums to learn about healthcare / research
(f) Other ________________________________

Section three: Importance of certain features of blogs that affect your engagement with them

(Directions: For the following questions, click the option that is closest to your opinion)

11. How important to you are the following aspects when considering reading a blog?

(a) Length of blog post
(b) Frequency of blog posts
(c) The author of the blog
(d) Use of images/infographics in blog
(e) Style of writing used in the blog post (personal vs third person)
(f) If you see the post being shared by a colleague
(g) If you see the post on a webpage
(h) If you see the post on twitter

Scale to use next to each option:

Not at all Important	Slightly Important	Important	Fairly Important	Very Important

12. **If we were to conduct monthly blog posts in the area of dementia palliative care research, would you read them?**

    (**Options:** Yes/No/Maybe
    Please explain? (comment box))

---

13. **If we were to conduct monthly blog posts in the area of dementia palliative care research, would you share them?**

    (**Options:** Yes/No/Maybe
    Please explain? (comment box))

---

14. **Any additional comments**
    (Option: comment box)

Thank you for completing this survey. As part of the Model for Dementia Palliative Care Project, we will soon begin adding a monthly blog post to our website about various health research topics. To keep up to date with our blogs and to learn more about our project please visit our website www.pallcare4dementia.com

If you have any questions about this research you may contact Dr Siobhan Fox by email s.fox@ucc.ie or phone +353 21 4627347
